# From Our Correspondents

**Published:** 1985-07

**Authors:** 


### From Our Corresponents

### Chair of Care of the Elderly

Frenchay Hospital has played a significant part in both undergraduate and postgraduate teaching for many years but the appointment of Professor Gordon Wilcock to the Chair of Care of the Elderly created by the University last year – and based on Frenchay – has increased Frenchay's teaching role still further. Four Undergraduates per week are seconded from firms at the B.R.I. for a week's course on various aspects of the care of the elderly, in addition to continuation of the previous teaching by many consultants and others in general medicine and general surgery.

Most doctors, with the limited exceptions of paediatricians and obstetricians (who may get the problems of the elderly at secondhand, so to speak), are liable to find the elderly form a large part of their work – and the role of a Department of Care of the Elderly is, among other things, to disseminate knowledge on how best to cope with such patients. The purpose of the new Academic Department is, in addition, to undertake research on the subject. Professor Wilcock's main research interest is Alzheimer's Disease – the all too prevalent brain rot – and he is a founder and Chairman of the Alzheimer's Disease Society, which raises funds and helps to co-ordinate research on this important subject, in the hope ultimately of being able to arrest the condition.

Professor Wilcock took a 1st Class Honours Degree in Anatomy at the London Hospital before graduating in medicine. He held an MRC Clinical Research Fellowship for training in research with Professor Sir Richard Doll in Oxford before being appointed Physician in Care of the Elderly at Oxford hospitals, with responsibilities also in acute medicine.This policy of dual training is to be continued in the new Department at Frenchay, where the Lecturer/Senior Registrar post in the Department has been accredited by the Royal College of Physicians for training in both geriatric and general medicine. This is part of an attempt to bring together general and geriatric medicine – as recommended by the Royal College of Physicians – without loss of identity of either. A classic example of co-operation rather than confrontation.

In that regard, most would agree that the evolution of specialisation in medicine (using the term in its widest sense) has too often been characterised by what might be termed 'keep-off-the grassitis' on the one hand and 'not-my-pigeonitis' on the other. Both terms are self-explanatory and imply regrettable attitudes towards colleagues. It has been well said that knowledge advances first by differentiation and then by integration. Both are essential to progress, but there have been occasions when the desire to continue differentiation has obscured the developing need to re-integrate – but in new ways. In other words to put the clock forwards – not to put it back to where it was before.

The overall policy of the Department has always been to concentrate on (a) prevention (keeping the elderly fit and in their own homes for as long as possible by treatment in Day Hospitals and other means) and (b) rehabilitation (since care of the elderly is as much about disability as about disease – although accurate diagnosis is essential). If these policies are actively and accurately pursued, the need for continuing care diminishes (although it will always remain to some degree).

The impending departure of Brandon Lush, who devloped the clinical service for the elderly on which the Academic Department is based, will allow for the further development of the Academic Department, since it is intended that he be replaced by a Consultant/Senior Lecturer. However, the remaining Consultant Physician in the Department, Dr. Ronald Barber, will continue to concentrate – as at present – on the service side, together with Professor Wilcock and the new Consultant, for it will be vital to ensure that the development of the academic side in no way diminishes the provision of a first-class clinical service for the elderly.

B. Lush

### Bristol 'Doctors of the Year'

The BUPA 'Doctor of the Year Award' was made this year to Mr. W. E. G. Thomas, a Senior Surgical Registrar at the Bristol Royal Infirmary. This award of £1000 is made annually by the BUPA Foundation and this year was presented by Princess Michael of Kent. Doctors under 40 are invited to submit research work published during the previous year and the award is made on the basis of scientific content, clinical relevance and clarity of presentation. Mr. Thomas submitted two papers, one on 'Sympathetic Orchidopathia' and the other on 'The possible role of duodeno-gastric reflux in the pathogenesis of gastric and duodenal ulcers'. The adjudicators unanimously awarded the distinction for the work on 'Sympathetic Orchidopthia', but the Governers of the Foundation said that under no circumstances could they speak about torsion of the testicle in front of HRH Princess Michael of Kent who was to present the Award, especially as Miss Moira Lister, the newscaster, was to read the citations. It seemed more socially acceptable to announce the award as for the work on Peptic Ulceration. And so it was officially decided. How quaint it seems that in these days of Women's Lib, explicit sex on TV that censorship should intrude on a scientific decision! How wise of Mr. Thomas to submit two papers. However, this was the time when the Princess was being subjected to emotional harrassment by the media over her father's connections with the SS. As it turned out this old-world courtesy was happily timed.

Mr. Bill Thomas acknowledges that he was put up to entering for the competition by his Senior Registrar colleague Mr. Michael Kelly, who himself won the title in 1981 for his work on Wound infection, and received his Award from Princess Alexandra.

The title 'Doctor of the Year' seems a little hyperbolic for an award which is somewhat limited in its scope – we seem to be entering the world of Oscars and Showbiz and no doubt at some future date will be seeing it on TV. Wishing not to end on a sour note one must say that Mr. Thomas's work is brilliant and of the first importance and one is glad to see it recognised.

M. G. Wilson

### Diploma in Geriatric Medicine

As many will be aware, the Royal College of Physicians has decided to add a new Diploma in Geriatric Medicine to the other postgraduate qualifications that it awards. This is not to be yet another examination for hospital doctors, but is rather intended to encourage a high standard of community care for the elderly. It appears that it will be aimed predominantly at general practitioners, and in many ways will resemble the DCH. This Diploma, which may also be appropriate for doctors opting for permanent sub-consultant appointments, will have its first examination in the late autumn/early winter of this year, and examiners and venues are currently being decided, if not already fixed by the time this appears in print. Readers will be pleased to know that one of the senior examiners has had strong connections with the South West for many years.

G. K. Wilcock

### Reduced State Support for Elderly in Private Nursing Homes

Of more immediate practical importance is the decision to reduce the resources available from 'State Funds' to support elderly people in private residential accommodation and nursing homes. It remains to be seen whether the fees will drop or if, as I believe to be more likely, there is going to be an increasing demand once again for hospital longterm care. In my opinion, the whole question of the most appropriate setting for the continuing care of our more dependent elders has been inadequately considered. We have a major responsibility to consider the dignity of their lives as well as the financial consequences of their care. They ask for little, and to remember that they are human beings rather than just statistics in a vast financial equation does not seem too unreasonable an expectation of our planners, both financial and otherwise.

G. K. Wilcock

### District General Managers in Bristol

Following the recommendations of the Griffiths report managers are being appointed at various levels within the Health Service. The appointment of a Regional General Manager was commented upon in the October 1984 edition of this Journal. General District Managers have now been appointed to two of the three Districts in Avon.

The Bristol and Weston General District Manager has already taken up office. He is Dr. John Roylance who is well known in the South West, having previously been a Consultant Radiologist and Director of the Department of Radiodiagnosis.

Dr. Roylance trained in Bristol and started his medical career as a house surgeon in the Bristol Royal Infirmary before taking a short commission in the R.A.M.C. He obtained the Montefiore Prize in Military Surgery but then opted for a career in Radiology. He returned to Bristol, initially as a senior registrar and then, from 1964, as a consultant.

During his time as a consultant John Roylance was very active in hospital management, being at various times chairman, treasurer, secretary or member of almost all of the committees ranging from the Hospital Medical Committee to the old Board of Governers and including lowlier gatherings such as the 'Drugs and Dressings Sub-Committee'. Nationally perhaps his most important administrative experience has been obtained as senior examiner for the Royal College of Radiologists and as Editor of the *British Journal of Radiology.*

Dr. John Roylance is internationally known and well respected as a uro-radiologist. Having taken on the new challenge of the Distict General Manager's post he is presently spending most of his time in a consultation period during which he is listening to people's problems and assessing the need for change. He sees this phase gradually shifting into a period of negotiation followed by implementation.

P. R. Godard

The Southmead District has appointed Mr. Bob Nichols to be General Manager. He is 45 and was previously assistant general manager with the South West Regional Authority. He is a former president of the Institute of Health Service Administrators. He started his Health Service career in Torquay in 1964 and moved to jobs in Southampton and Newcastle before joining the South West Region 4 years ago.

He recently gave a talk at Southmead Postgraduate Centre to medical staff and spoke on the role of the district manager as he saw it. Until the changes brought about by the Griffiths Report the ultimate accountability for everything that happened in the Health Service rested in the Minister. As it is manifestly impossible for one man to be aware of every development it is a meaningless concept and really no-one was responsible, especially in the preceding administration by consensus by management teams. He saw the Manager's role as to secure agreed changes by knowing where to go and how to go – without going round in circles in endless committees, to squeeze more out of the money you have and to see that options are developed. The Southmead District is the biggest local employer of labour with a staff of 4000 and an income of £45,000,000. He has already established a close liaison with the medical staff and given the impression that an era of fruitful cooperation is about to begin.

M. G. Wilson

**Down with Decibels**

I have always thought that the road signs outside hospitals saying 'Quiet Please' were an anachronism. By far the greatest amount of noise is produced within. Every year in the hospitals which I serve, there seems to be the need for pneumatic drills near my place of work, and powerful electric drills in walls near my wards. The start of my ward round is a signal for the electric polisher to work flat out, for nurses to drop bowls, trays and bed pans and for loud conversation in adjacent beds. When I was once warded, the nights were disturbed by bed pan washing machines, noisy trolleys and night nurses enjoying loud chats in the small hours. In hospitals for the elderly or mentally ill, the radio or television seems constantly to be numbing patients'brains.

In the last century, we have conquered most food- and water-borne disease. The air we breathe is now much purer than ever before, but have we considered enough the ill-effects of excessive noise on the nervous system? We know that loud pop music and working in noisy machine shops can cause premature deafness. Deaf people are commonly very tranquil as they do not have to put up with excessive auditory stimuli which seem to accompany 'civilisation'. Many patients have told me recently how a noisy environment has made their treatment in hospital a survival course which they reluctantly endure. I put in a plea for us to restrict noise in our places of work. One of the most important milieux for smooth convalescence whether from a coronary or a major operation is peace and quiet. Down with decibels!

H. G. Mather

**Hospital Blight**

What is it about hospitals? They are usually busy places with plenty of people coming and going each day. Many are established in the decaying centre of towns and cities (much to the consternation of anyone organising a meeting and in need of parking facilities); others have left the to-and-fro of urban life and have taken abode in the distant suburbs.

None the less, dare one say it, they all seem to have a strong sort of B.O. which is especially evident to neighbouring commercial ventures. To a naïf it is amazing that so few shops seem to thrive around today's hospitals. Why should this be so? Those of you who read books on urban development will know the stock answers. Property values and access are imperilled by the presence of hospitals. Not unnaturally they, like Universities, Water Works and Railways, are then tempted to buy in their neighbourhood. One can easily imagine the result.

Why doesn't anyone try to reverse the trend? Comers and goers – relatives or patients – are still people. Wouldn't shopping arcades, the odd supermarket or chain store benefit from the association? Suppose one were to think of building a hospital in the middle of a shopping centre. Even our masters at the Department of Health and Social Security in the Elephant & Castle have managed that. Why not move Barts to Oxford Street and Frenchay to Broadmead? It certainly ought to improve follow-up attendances. Even those earnest souls coming to meetings might welcome the shopping.

J. D. Davies

